# Age-Related Differences in Experiences With Social Distancing at the Onset of the COVID-19 Pandemic: A Computational and Content Analytic Investigation of Natural Language From a Social Media Survey

**DOI:** 10.2196/26043

**Published:** 2021-06-09

**Authors:** Ryan C Moore, Angela Y Lee, Jeffrey T Hancock, Meghan C Halley, Eleni Linos

**Affiliations:** 1 Department of Communication Stanford University Stanford, CA United States; 2 Center for Biomedical Ethics Stanford University Stanford, CA United States; 3 Department of Dermatology Stanford University Stanford, CA United States

**Keywords:** COVID-19, natural language processing, public health messaging, social distancing compliance, age differences, older adults, younger adults, age, NLP, public health, elderly, youth, adult, emotion, compliance, guideline

## Abstract

**Background:**

As COVID-19 poses different levels of threat to people of different ages, health communication regarding prevention measures such as social distancing and isolation may be strengthened by understanding the unique experiences of various age groups.

**Objective:**

The aim of this study was to examine how people of different ages (1) experienced the impact of the COVID-19 pandemic and (2) their respective rates and reasons for compliance or noncompliance with social distancing and isolation health guidance.

**Methods:**

We fielded a survey on social media early in the pandemic to examine the emotional impact of COVID-19 and individuals’ rates and reasons for noncompliance with public health guidance, using computational and content analytic methods of linguistic analysis.

**Results:**

A total of 17,287 participants were surveyed. The majority (n=13,183, 76.3%) were from the United States. Younger (18-31 years), middle-aged (32-44 years and 45-64 years), and older (≥65 years) individuals significantly varied in how they described the impact of COVID-19 on their lives, including their emotional experience, self-focused attention, and topical concerns. Younger individuals were more emotionally negative and self-focused, while middle-aged people were other-focused and concerned with family. The oldest and most at-risk group was most concerned with health-related terms but were lower in anxiety (use of fewer anxiety-related terms) and higher in the use of emotionally positive terms than the other less at-risk age groups. While all groups discussed topics such as acquiring essential supplies, they differentially experienced the impact of school closures and limited social interactions. We also found relatively high rates of noncompliance with COVID-19 prevention measures, such as social distancing and self-isolation, with younger people being more likely to be noncompliant than older people (*P*<.001). Among the 43.1% (n=7456) of respondents who did not fully comply with health orders, people differed substantially in the reasons they gave for noncompliance. The most common reason for noncompliance was not being able to afford to miss work (n=4273, 57.3%). While work obligations proved challenging for participants across ages, younger people struggled more to find adequate space to self-isolate and manage their mental and physical health; middle-aged people had more concerns regarding childcare; and older people perceived themselves as being able to take sufficient precautions.

**Conclusions:**

Analysis of natural language can provide insight into rapidly developing public health challenges like the COVID-19 pandemic, uncovering individual differences in emotional experiences and health-related behaviors. In this case, our analyses revealed significant differences between different age groups in feelings about and responses to public health orders aimed to mitigate the spread of COVID-19. To improve public compliance with health orders as the pandemic continues, health communication strategies could be made more effective by being tailored to these age-related differences.

## Introduction

A signature of the COVID-19 pandemic is that the virus poses different levels of threat to individuals of different ages. In the United States, nearly three-quarters of all deaths attributable to COVID-19 have occurred in individuals ≥65 years of age [1]. By contrast, 4% of total deaths have been in individuals ≤34 years, and 22% have been in individuals between 35-64 years. As such, recent evidence suggests that older and younger individuals may differ substantially in their behavioral and attitudinal responses to COVID-19 [2]. For instance, younger people may be more likely to engage in activities that increase the risk of virus transmission, such as dining indoors or attending social gatherings, than older people.

We fielded a survey on social media early in the pandemic just as the first state-issued shelter-in-place orders were implemented in order to understand how different age groups experienced the impact of the coronavirus crisis and the extent to which they complied with self-isolation mandates. Specifically, we sought to understand older and younger peoples’ experiences with the pandemic through analysis of those groups’ use of language—that is, the text of their responses to open-ended survey questions. Prior work has shown that human language can provide a rich profile of how people are feeling about and experiencing daily life [3].

Understanding the public’s experiences through language analysis may be particularly valuable during times of rapid change and crisis [4]. Researchers have previously analyzed language to understand how individuals are experiencing and responding to unprecedented situations, such as the present pandemic. For example, Cohn et al [5] analyzed language in online journal entries before and after the September 11, 2001, terrorist attacks in the United States and uncovered pronounced psychological changes in response to the attacks. By examining the sentiment of language and pronoun usage using computational methods, the authors found that individuals expressed more negative emotions and were less self-focused in the 2 weeks following 9/11. In addition, the analysis revealed that individuals varied considerably in the extent to which they discussed the events of 9/11 and related topics.

In this paper, we use language to explore two core questions regarding how different age groups are responding to the COVID-19 pandemic. First, how do different age groups experience the impact of the pandemic? While the pandemic is having broad-reaching effects on nearly all parts of our lives, different age groups may focus on different aspects or experience different emotions. We examine how older and younger individuals, who face distinct levels of health risk from the virus, differentially experience those effects.

Building off the work of Cohn et al [5] on individuals’ experiences of 9/11, we specifically wanted to explore 3 dimensions of language to examine differences in experiences between age groups:

1. Sentiment: Do older individuals express more positive or negative affect regarding their experience with the virus? Prior research has demonstrated that older individuals tend to be more emotionally positive than younger people [6,7], but with the increased risk from the pandemic, will this trend toward positivity persist?
2. Self vs other focus: Do different age groups, given differing levels of personal risk, vary in the extent to which they focus on themselves compared to others when discussing the virus?
3. Topical salience: Some virus-related topics may be more salient for some age groups than others (eg, health care or symptoms are more important for older than younger individuals).

More formally, we ask the following research question (RQ):

RQ1. How do age groups differ in how they use emotional language, self-other focus, and topical salience when describing their experience during the onset of the pandemic?

Given their differential levels of health risk, we also examined whether individuals of different age groups differed in noncompliance with the health mandates of social distancing and self-isolation. Reporting on the pandemic has drawn attention to COVID-19 clusters caused by noncompliance among communities of different ages, such as outbreaks linked to parties on college campuses and large gatherings at events like weddings [8,9]. In addition to obtaining base rates of noncompliance by age group by surveying participants about their adherence to health mandates, analyzing the language from individuals’ open-ended responses allows us to explore the specific reasons people provide for not complying more often with social distancing recommendations, which have been demonstrated to be effective at slowing the spread of viral infections such as COVID-19 [10,11]. More formally, we ask the following:

RQ2. What were the rates and reasons for noncompliance with social distancing guidelines at the onset of the pandemic by age group?

In the context of public health messaging, understanding the language individuals use to describe their health-related thoughts, feelings, and actions is essential to developing effective, scalable communication strategies for different groups who may face different levels of risk or who may behave differently in the face of a major health episode such as a pandemic [12,13]. As can be seen by increasing numbers of young people breaking social distancing protocols, more tailored interventions may be needed to communicate more effectively with individuals at different levels of health risk. Understanding how the public conceptualizes and experiences the COVID-19 health threat is crucial for public health measures requiring citizens to comply with unprecedented behavioral changes. The goal of this paper is to explore age-related differences in the experience of the pandemic (RQ1) and in peoples’ noncompliance with COVID-19 prevention measures (RQ2).

## Methods

### Recruitment

We recruited a convenience sample of individuals impacted by the COVID-19 pandemic by posting our survey online from March 14-23, 2020. In order to maximize responses, we posted our call for respondents on Twitter, Facebook, and Nextdoor. Upon seeing recruitment materials for our study on social media, individuals could elect to participate in our study. The survey included a total of 21 questions including demographics, the impact of COVID-19 on individuals’ daily life, actions taken in regard to COVID-19, and difficulties faced related to the pandemic [14]. The study was approved by the authors’ institution’s Institutional Review Board.

### Statistical Analysis

Our first research question examined how individuals’ language about their experience with the pandemic, including expressed sentiment, self or other focus, and topical salience, differed across age groups. To do this, we analyzed open-ended responses to the survey-question, “Tell us how the coronavirus crisis is impacting your life” using Linguistic Inquiry and Word Count (LIWC), a well-validated and widely used computerized text analysis program [15]. LIWC counts the number of words in a variety of psychological (eg, positive or negative emotion terms), topical (eg, family-related terms, work-related terms), and part of speech (eg, pronouns, adverbs) categories that appear in a given text relative to all the words in that text. To further explore topical focus in people’s descriptions of the impact of COVID-19, we identified themes in open-ended responses for each age group using the meaning extraction method, which relies on principal component analysis (PCA) of content words in language corpuses [16]. Data were processed with the Meaning Extraction Helper software to remove function words (ie, prepositions) and words with low base rates (present in <5% of responses), and calculate whether content words (ie, nouns, verbs) were present (coded as “1”) or absent (coded as “0”) within a response [17]. We then conducted separate PCAs on the responses for each of the four age groups.

Our second research question explored rates and reasons for noncompliance with social distancing and isolation orders by age group. Overall rates of compliance and noncompliance were calculated by examining responses to questions asking whether participants were social distancing and isolating as much as possible. To investigate the reasons for noncompliance, participants were asked to select from a list of preselected reasons (eg, not being able to miss work), with the option to write in another reason. We conducted a thematic content analysis to identify, analyze, and report themes in these responses (Multimedia Appendix 1). This process was conducted by 2 independent raters with good interrater reliability (Cohen κ=0.76-0.81).

## Results

### Participant Demographics

We collected a total of 17,687 responses in 9 days. We excluded 400 individuals from our data set who did not provide information on age as this was integral to all of our analyses. Thus, the resulting data set consisted of 17,287 individuals. The mean age of the sample was 45.5 years, with 16.4% (n=2905) of the sample aged 18-31 years, 34.2% (n=6054) aged 32-44 years, 36.3% (n=6417) aged 45-64 years, and 10.8% (n=1911) aged ≥65 years. These age groups are modeled after the age groups reported by the Center for Disease Control and Prevention in their summary of COVID-19 cases in the United States [18]. The majority of respondents identified as White (14,340/17,287, 83%) and were located in the United States (13,183/17,287, 76.3% provided a valid US zip code when asked). In addition, the sample was relatively highly educated (high school diploma or less: 426/17,287, 2.5%; some college: 2444/17,287, 14.1%; bachelor’s degree: 5273/17,287, 30.5%; graduate degree: 9132/17,287, 52.8%; no information provided on educational background: 12/17,287, 0.07%).

### RQ1: Language and the Impact of the COVID-19 Pandemic

**Figure 1:**
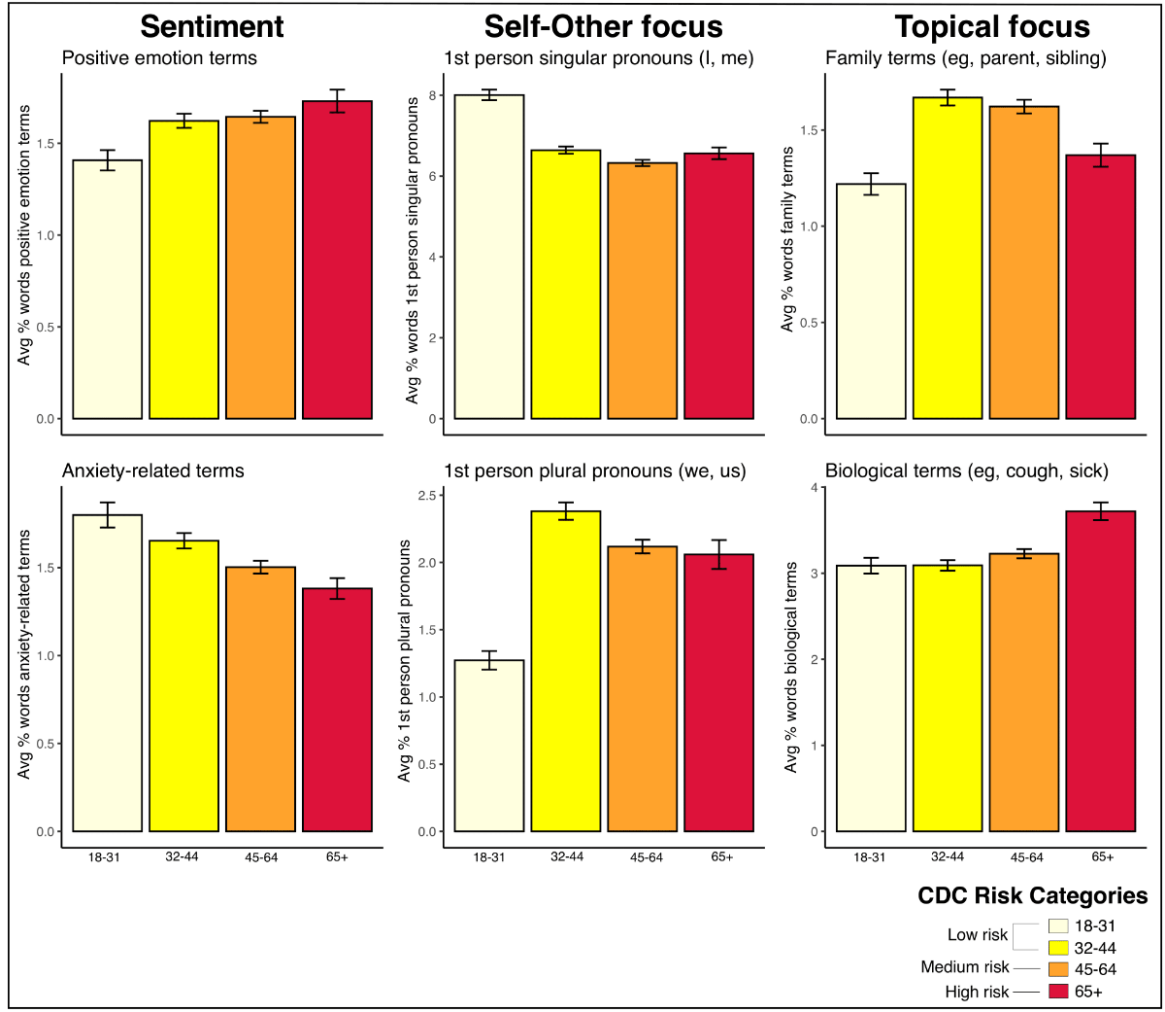
Mean number of words in language categories, by age group. Age groups are modeled after the age groups reported by the Centers for Disease Control and Prevention (CDC) in their summary of COVID-19 cases in the United States [18]. Bars represent standard errors.

Of the 17,287 total survey responses, 6573 individuals provided a response ≥30 words to the open-ended question “Tell us how the coronavirus crisis is impacting your life.” This length cutoff was used since the LIWC development manual suggests a minimum of at least 25 words [15]. As Figure 1 shows, younger people (18-31 years) were more anxious (greater usage of anxiety-related terms), less emotionally positive (lesser usage of positive emotion terms), self-focused (greater use of first-person singular pronouns), and less concerned with family (lesser use of family-related terms), while middle-aged people were group-oriented (32-44 years; greater use of first-person plural pronouns) and focused on family (32-64 years; greater use of family-related terms). Unsurprisingly, the oldest and most at-risk group (≥65 years) wrote frequently about biological terms (eg, health-related topics) but were surprisingly low in anxiety (use of fewer anxiety-related terms) and emotionally positive (greater use of positive emotion terms) relative to those at lower risk (all *P* values corrected for multiple comparisons; see Multimedia Appendix 1 for the results of relevant age group comparisons).

### RQ1: Topical Salience in Describing the Impact of COVID-19

The extraction of qualitative themes from participants’ responses through the meaning extraction method allowed us to gain deeper insight into what topics people of different age groups focused on during the pandemic onset. For each of the five factors analyzed, content words were retained if their loadings were over or equal to the absolute value of .30 [16]. As seen in Table 1, people of different age groups focused on distinct aspects of their experiences. Some topics, such as acquiring essential goods and supplies (ie, groceries) and engaging in COVID-19 prevention behaviors (ie, social distancing, hand washing), were important for people of all age groups. Other themes, however, were specific to certain age groups. The youngest age group wrote about the impact of school closures and moving home, the middle age groups wrote about the impact of COVID-19 on work and family, and the oldest age group wrote about being at high risk for COVID-19 and engaging with community services. In addition, while all groups discussed the impact of limited social interactions, young people focused on the process of adjusting, middle-aged people focused on canceled trips, and older people focused on canceled activities with family.

**Table 1:** Results of the principal component analysis for open-ended responses about the impact of the pandemic by age groups. Note: columns denote component numbers extracted from separate principal component analyses for each age group, subjected to Varimax rotations. Words were selected for inclusion on the component if their loading was greater than or equal to |.30|.

Age group		Components				
		1	2	3	4	5
**18-31 years**						
	Theme	Concern for family	School closure	Essential supplies	Social distancing effects	Compliance
	λ, % variance	2.88, 3.0	2.78, 3.0	2.68, 3.0	2.61, 3.0	2.29, 2.0
	Word (loading)	Family (.42) Worry (.39) Sick (.39) Member (.39) Health (.37) Virus (.36) Care (.34) Due (.31) Risk (.30} Mother (.30)	Class (.69) Online (.69) Student (.48) School (.43) Cancel (.43) College (.43) Move (.42)	Store (.51) Grocery (.47) Supply (.44) Shop (.33) Clean (.33) Place (.31) Close (.31) Stock (.30)	Distance (.45) Social (.42) Walk (.41) Friend (.34) Hard (.33) Isolate (.33) Leave (.32)	Wash (.64) Hand (.63) Social (.47) Distance (.36)
**32-44** **years**						
	Theme	COVID-19 spread	Essential supplies	Impact on work and family	Social distancing and canceled plans	School closure
	λ, % variance	2.84, 3.0%	2.83, 3.0	2.39, 3.0	2.29, 2.0	2.11, 2.0
	Word (loading)	Test (.61) Symptom (.50) COVID (.45) Sick (.42) Hospital (.33) People (.31) Health (.31)	Hand (.56) Grocery (.55) Store (.54) Wash (.47) Food (.46) Shop (.37) Supply (.36) Stock (.35) Clean (.35)	School (.56) Husband (.47) Work (.45) Kid (.39) Week (.37) Close (.37) Cancel (.33) Child (.30)	Social (.53) Distance (.44) Family (.36) Cancel (.35)	Online (.66) Class (.61) College (.55) Move (.50)
**45-64** **years**						
	Theme	School closure and family	Essential supplies	COVID-19 spread	Social distancing and canceled plans	Concern for family
	λ, % variance	2.75, 3.0	2.75, 3.0	2.57, 3.0	2.31, 2.0	2.27, 2.0
	Word (loading)	School (.60) Online (.54) College (.54) Class (.49) Student (.37) Daughter (.36) Move (.35) Close (.32) Husband (.31) High (.31) Son (.31)	Food (.57) Supply (.52) Store (.43) Hand (.43) Stock (.41) Clean (.40) Grocery (.38) Wash (.35) House (.33)	Test (.50) Symptom (.48) COVID (.48) Sick (.36) Case (.30)	Social (.60) Distance (.50) Cancel (.44) Plan (.36) Trip (.36) Activity (.31)	Worry (.39) Elderly (.36) Live (.35) Parent (.35) Concern (.33) Health (.31) Hand (–.31) Wash (–.34)
≥**65** **years**						
	Theme	Essential supplies	Community concerns	Social distancing and canceled events	High-risk status	Supporting family
	λ, % variance	2.89, 3.0	2.69, 3.0	2.56, 3.0	2.22, 2.0	2.22, 2.0
	Word (loading)	Hand (.55) Wash (.54) Grocery (.48) Store (.47) Clean (.40) Shop (.30)	Student (.35) Community (.33) Member (.32) Hospital (.32) Small (.31) Day (.31) Class (.30)	Cancel (.52) Family (.52) Social (.47) Plan (.43) Activity (.36) Event (.35) Trip (.32) Time (.31)	Risk (.57) High (.49) Virus (.39) Sick (.35)	Supply (.48) Food (.46) Mother (.38) Job (.35) Visit (.32) Find (.30)

### RQ2: Rates of Compliance and Reasons for Noncompliance

Although 10,782 participants said they were not complying with social distancing and isolation orders as much as possible in a closed-ended question, analysis of their written open-ended responses revealed that approximately 30% (n=3326) were in fact in compliance with recommended health guidelines (ie, only leaving their homes to buy groceries, find essential supplies, or attend necessary medical appointments). Compliance with guidance was based on the initial guidelines present during the time of data collection, which were published just ahead of the first stay-at-home order in California (March 19, 2020) [19]. Thus, out of 17,287 survey responses, 43.1% of participants reported not fully complying (n=7456) with shelter-in-place orders. A large number of respondents (n=7416, 42.9% of total respondents) provided a reason of their own for noncompliance.

We then explored the reasons why participants did not fully comply with COVID-19 prevention guidelines, such as social distancing and self-isolation (Table 2). Of those who were noncompliant, the most common reason reported was not being able to miss work (4273/7456, 57.3%). Other reasons for not complying with health orders included not having sufficient space to self-isolate (719/7456, 9.6%), meeting mental and physical health needs (533/7456, 7.1%), feeling that other precautions were sufficient (eg, frequent handwashing; 488/7456, 6.5%), wanting to continue engaging in nonessential activities (366/7456, 4.9%), feeling that society was overreacting (339/7456, 4.5%), not believing social isolation was effective at preventing the spread of COVID (281/7456, 3.8%), needing to attend classes in person (180/7456, 2.4%), and concerns about caring for children in isolation (129/7456, 1.7%).

**Table 2:** Reasons for noncompliance with COVID-19 health orders by age group. Percentages were calculated as a proportion of noncompliant individuals in each age group.

Theme	Example	Total noncompliant (n=7456), n (%)	Age category			
			18-31 years (n=1589), n (%)	32-44 years (n=2668), n (%)	45-65 years (n=2653), n (%)	≥65 (n=546), n (%)
Cannot afford to miss work^a,b^	“Work is not canceled, if I don’t go I’ll lose my job.”	4273 (57.3)	949 (59.7)	1689 (63.3)	1489 (56.1)	146 (26.7)
Mental and physical health needs^b^	“Total self-isolation would probably drive me to suicide.”	533 (7.1)	145 (9.1)	169 (6.3)	167 (6.3)	52 (9.5)
Taking sufficient precautions^b^	“I already wash my hands regularly and cover my mouth when I cough or sneeze. I am not concerned with catching [the] virus.”	488 (6.5)	50 (3.1)	115 (4.3)	204 (7.7)	119 (21.8)
No space to self-isolate^a^	—^c^	719 (9.6)	293 (18.4)	246 (9.2)	150 (5.6)	30 (5.5)
Nonessential activities^b^	“Some appointments are in-person. Need to see friends sometimes.”	366 (4.9)	72 (4.5)	91 (3.4)	156 (5.9)	47 (8.6)
Society is overreacting^b^	“I think the news media was making everyone panic and overreact.”	339 (4.5)	46 (2.9)	95 (3.6)	152 (5.7)	46 (8.4)
Do not believe social isolation to be effective^c^	—	281 (3.8)	61 (3.8)	92 (3.4)	96 (3.6)	32 (5.9)
Kids^b^	“Really hard to do with little kids - I’m reducing a lot of contact, but not all.”	129 (1.7)	6 (0.4)	79 (2.9)	39 (1.5)	5 (0.9)
Have to attend in-person classes^c^	—	180 (2.4)	86 (5.4)	42 (1.6)	44 (1.6)	8 (1.5)

^a^Indicates that this theme was identified through participants’ responses to a multiple-choice question.

^b^Indicates that this theme was identified through thematic content analysis of participants’ text responses.

^c^For themes only identified through multiple-choice questions, no example response is available.

We then examined how noncompliance rates and reasons varied by age group. A chi-square test of noncompliance by age group was significant (χ^2^_3, 17,283_=113.56, *P*<.001) and revealed that noncompliance decreased with age. The youngest group (18-31 years) had the highest rate of noncompliance while the oldest age group had the lowest. People of different age groups also differed in their reasons for noncompliance, including work (χ^2^_3, 17,283_=150.11, *P*<.001), mental and physical health needs (χ^2^_3, 17,283_=14.34, *P*<.001), feeling like other precautions were sufficient (χ^2^_3, 17,283_=38.70, *P*<.001), not having space to self-isolate (χ^2^_3, 17,283_=116.17, *P*<.001), wanting to participate in nonessential activities (χ^2^_3, 17,283_=5.66, *P*=.001), believing that society was overreacting (χ^2^_3, 17,283_=4.84, *P*=.002), concerns about kids (χ^2^_3, 17,283_=14.92, *P*<.001), and having to attend classes in person (χ^2^_3, 17,283_=42.30, *P*<.001). Frequencies of reasons for noncompliance by age group can be found in Table 2.

The pattern of results suggest that while work obligations proved challenging for participants across ages, younger people struggled more to find adequate space to self-isolate and manage their mental and physical health, middle-aged people faced more concerns regarding childcare, and older people perceived themselves as able to take sufficient precautions. Our results provide important insights into why different people fail to comply with COVID-19 prevention measures like social distancing.

## Discussion

### Principal Findings

Our findings from a survey of thousands of Americans early in the pandemic (March 14-23, 2020) reveal important age-related differences in how people experienced the impact of COVID-19 at the outset of the pandemic (RQ1) and in the extent to which they complied with social distancing and self-isolation orders (RQ2).

As discussed in Cohn et al [5], language can provide insight into how people are thinking and feeling during times of crisis. Examining how people of different risk levels experienced the impact of the COVID-19 pandemic and their reasons for noncompliance can inform communication and interventions to increase compliance across the board while recognizing the unique needs of individuals from different age groups. We found that, in discussing the impact of COVID-19, younger individuals were more emotionally negative and self-focused, while middle-aged people were other-focused and concerned with family. The oldest and most at-risk group was most concerned with health-related terms but were also lower in anxiety and higher in the use of emotionally positive terms than the other, less at-risk age groups. PCA-driven topical analyses in participants’ description of the impact of the pandemic on their lives supported these age-related differences. While all groups discussed necessary lifestyle changes caused by COVID-19, such as acquiring essential supplies, individuals of different age groups wrote about the impact of school closures and limited social interactions in different ways.

We also found relatively high rates of noncompliance with COVID-19 prevention measures, such as social distancing and self-isolation. However, like emotional experiences of the pandemic, rates of noncompliance varied significantly by age group. While 7456 of 17,287 respondents (43.1%) reported that they were not isolating as much as recommended, this number appears to be driven by higher rates of noncompliance among younger and middle-aged people. The youngest age group (18-31 years) had the highest rate of noncompliance, with more than half of respondents (1589/2905, 54.7%) reporting they did not isolate sufficiently. Middle-aged adults had lower rates of noncompliance (32-44 years: 2668/6054, 44.1%; 45-65 years: 2653/6417, 41.3%). The oldest age group, which faced the highest level of health risk from COVID-19, was the most compliant, with only 546 of the 1911 respondents aged ≥65 years not fully following COVID-19 health orders (28.6%).

We also advanced our understanding of why people were not or could not comply with health orders. Our results suggest reasons for noncompliance were nuanced and varied. The predominant reason given for not being able to follow social distancing and self-isolation orders was not being able to afford to miss work. Of the respondents who were not isolating as much as recommended, more than half listed work as the reason. Some participants indicated they were essential service workers or health care professionals; however, others working in nonessential industries also reported that work obligations and conditions prevented them from social distancing and self-isolating more. Future public health communications encouraging compliance with existing health guidance should be targeted not only at individuals but also at employers on how to minimize COVID-19 exposure, prevent viral spread in the workplace, and protect individuals working during the pandemic.

Age-related differences in noncompliance reflect how each group experienced the pandemic, and these differences can inform future health communication strategies to enhance compliance to public health orders. We describe key health communication strategies by age group in Table 3. People in the youngest age group (18-31 years) were the most likely to say they could not fully comply with health guidance because they did not have sufficient space to self-isolate (293/2905, 10.1%). Given the prevalence of shared residences (eg, college dorms, apartments) among young adults, this could be addressed through community-specific health messaging that provides guidance on how to minimize COVID-19 spread within shared living spaces. The youngest age group was also the most likely to be noncompliant because of the detrimental impact of self-isolation and social distancing on their mental and physical health. In discussing their experience with the pandemic, they were highly negative, expressing significantly more anxiety and using less positive emotion terms relative to the other age groups, and focused on the process of adjusting to limited social interactions. Together with results from the COVID Response Tracking Study that the majority of young Americans aged 18-34 years are experiencing poor mental health [20], these findings underscore the need for health communications targeting this age group to be responsive to the emotional impact of the pandemic on their lives. Future interventions should publicize information on available mental health resources and provide guidance on how to take care of mental health needs while complying with health orders.

People in the middle-aged groups were predominantly noncompliant because they could not afford to miss work. This age group was unique in that they were primarily focused on family. When they described the impact of the pandemic on their lives, they used the most first-person plural pronouns (ie, we, us), suggesting a group-oriented (as opposed to self-focused) mindset, and were the most likely to use language related to family. In addition, they focused on the impact of the pandemic on work and school closures for their family. Almost all of the individuals who cited children and childcare as a reason for noncompliance with health orders were in this age group. As more research emerges on the challenges of parenting and caring for others during the pandemic, health communications targeting these age groups should discuss strategies to maintain social distance while caring for children and family members. In addition, such messages should remind those who are caring for others to take care of themselves.

**Table 3:** Health communication strategies for COVID-19 messaging by age group.

Age group	Experience of the pandemic	Noncompliance reasons	Individual-level messaging recommendations	Institution-level messaging recommendations
18-31 years	Highest in anxiety and lowest in positive emotion terms Most focused on themselves	Most likely to cite mental health toll Most likely to cite need to work and to attend school Most likely to cite not having sufficient space to self-isolate	Address negativity by focusing on positive future outlook Emphasize the consequences of their virus-related behaviors on other people Publicize information about available mental health resources and share advice on how to take care of one’s mental health needs while complying with health orders Discuss how to stay safe while at work (eg, wear masks during breaks), at school (eg, sanitize books and computers), or while exercising (eg, maintaining social distancing while running) Provide guidance on minimizing COVID-19 spread within a shared living space (ie, college dorms, apartments)	Provide guidance on minimizing COVID-19 spread within a shared living space (ie, college dormitories, apartments) Discuss how to stay safe while at work (eg, wear masks during breaks), at school (eg, sanitize books and computers), or while exercising (eg, maintaining social distancing while running, prioritizing outdoors exercise) Institutions should clearly communicate the importance of prevention measures for both personal and collective health
32-64 years	Most focused on others Highly focused on family	Most likely to cite childcare as reason for noncompliance	Remind those caring for others to care for themselves	Provide strategies for how to safely social distance while caring for kids or other family members
≥65 years	Most focused on health-related terms Lowest in anxiety and highest in positive emotion terms	Most likely to say they’re already taking sufficient precautions	Recognize efforts and precautions already being taken by older populations Discuss symptomatology of the virus and provide clear instructions for accessing health services Provide information and resources to improve quality of life in isolation	Discuss strategies for how to safely social distance while caring for kids or other family member Discuss symptomatology of the virus and provide clear instructions for accessing health services Provide information and resources to improve quality of life in isolation

It is perhaps not surprising that the oldest age group was the most likely to say they were complying as much as possible with health guidelines (1365/1911, 71.4%) given their elevated risk to the virus. In addition to focusing on their high-risk status in discussing the impact of the pandemic on their lives, they were also most likely to use health terms relating to sickness and symptoms. Surprisingly, however, they appeared to be resilient to the negative emotional effects of COVID-19—using the fewest number of words relating to anxiety and the most words relating to positive emotion to describe the impact of the pandemic on their lives. These results support suggestions made by others that, despite COVID-19 presenting a great deal of health risk to older adults, older adults possess life experience, perspectives, and contexts that can help them be emotionally positive and resilient in the face of the pandemic [21,22]. As the pandemic continues, health communications should be cognizant of the precautions already taken by older populations to keep themselves safe and recognize their ongoing efforts; rather than focusing on telling them to do things that most are already doing diligently, messaging should provide guidance on how to improve their quality of life while they continue self-isolation.

The increased emotional positivity and reduced self-focus in the language of older adults relative to younger adults has been documented in prior work examining large corpora of natural language generated by individuals across the lifespan in a diversity of contexts [23]. Given the elevated risk to older adults posed by COVID-19, one might expect the patterns of greater emotional positivity and reduced self-focus to disappear. Instead, we find that when asking specifically about the impact of the coronavirus crisis, the language of older adults still tends to be more emotionally positive and less self-focused than that of younger individuals. Consistent with other recent work [21], these results provide evidence that older adults’ emotional positivity and reduced self-focus is robust even in the face of a significant threat.

While the general pattern of older individuals being more positive than younger individuals holds in the context of the pandemic, this positivity bias may be attenuated. We used data from two prior large-scale studies of natural language [23,24] to explore the extent to which the size of the gap in positive language usage between old and young adults observed in our data was comparable in size to gaps in non–COVID-19–related language (see Multimedia Appendix 1 for details on these comparisons). We found that the emotional positivity bias during COVID-19 was significantly smaller in magnitude than that bias observed in non–COVID-19 language (about 3 times smaller than the bias in Pennebaker and Stone [23] and 1.2 times smaller than the bias in Schwartz et al [24]). Furthermore, the difference we observed in anxiety-related words between older and younger adults (with younger people displaying significantly greater anxiety) was significantly larger in magnitude than in the non–COVID-19 language from prior works (about 7.8 times larger than the bias in Schwartz et al [24]). These exploratory comparisons suggest that the pandemic is exerting strong influences on different age groups’ emotions, which are being reflected in their language. The well-documented positivity bias in the emotions of older adults was observed here, but the size of that bias shrank during COVID-19, possibly because the pandemic poses a significant new threat to the health of older individuals.

Of the seniors who said they were not fully compliant with social distancing and self-isolation measures (546/1911, 28.6%), they were most likely to say this was because they could not afford to miss work (146/546, 26.7%) or because they felt like they were taking sufficient other precautions outside of the health guidance (ie, frequent hand washing, generally avoiding people but not social distancing or isolating) (119/546, 21.8%). In response to the latter issue, health messaging should provide clear, consistent reminders about what constitutes sufficient “compliance” with COVID-19 prevention measures, particularly as health orders change throughout the pandemic.

The ability to rapidly assess public sentiment through natural language processing can facilitate informed policy decision making during a pandemic. Natural language processing methods such as LIWC and the meaning extraction method allow researchers, policymakers, and government officials to “take the pulse” of their citizens, to see how they are experiencing the impact of the pandemic, and to know why they are or are not complying with public health orders. Such insights may help legislators and health strategists pivot their messaging to be more responsive to the needs of the public and tailored to the challenges facing specific communities.

### Limitations

There are several limitations to our research. First, our use of an online convenience sample and recruitment via social media may have potentially influenced the characteristics of our sample [25]. Of note, our sample is especially highly educated and comprised more White individuals relative to the broader United States population. Future work on age-related differences in COVID-19 experiences and noncompliance should involve nationally representative data and lifespan sampling to have a more representative sample from which conclusions can be more generalizable. It is worth noting, however, that our finding that older individuals are emotionally positive in the face of COVID-19 is corroborated by recent findings collected from a nationally representative sample [21]. In addition, we may observe some degree of social desirability in our responses given that we are asking about a socially charged issue. While we did observe a relatively high rate of noncompliance in our study, it may be that additional people who were noncompliant were not willing to admit it. Furthermore, we may see biases in the reasons participants give for their noncompliance, such that participants may be less willing to report that they are failing to fully comply with health orders because of reasons that are socially undesirable, such as wanting to go to social engagements.

Our timing of data collection early in the COVID-19 outbreak in the United States may mean that participants’ experiences look different now than they did earlier given the rapidly changing nature of the pandemic. Continuous assessment of public sentiment and responses to health guidance is necessary to understand current experiences as the circumstances of the pandemic change over time. Finally, regarding our public health messaging recommendations, while age is a key demographic characteristic upon which health communication messages can be tailored, personalization of communications using multiple demographic and behavioral characteristics has been found most effective in inciting behavior change [26]. Policymakers and other communicators should consider multiple characteristics when designing messages around COVID-19 (eg, age, gender, socioeconomic status, health status).

### Conclusions

Our findings suggest that there are meaningful differences in how people of different ages experience COVID-19 and respond to health measures to prevent its spread, such as social distancing. Notably, younger people (18-31 years) discussed the impact of COVID-19 with more self-focused and negative emotional language, middle-aged people were more other-focused (32-44 years) and concerned with family (32-64 years), and older people (≥65 years) were more concerned with health-related terms but were also lower in anxiety. Despite the threat posed to older people by COVID-19, they were more emotionally positive than young people in their language use. However, we present evidence that the magnitude of this positivity bias may be attenuated by the pandemic. A closer examination of noncompliance with COVID-19 prevention measures also revealed age-related differences. Although the most common reason for noncompliance across age groups was not being able to afford missing work, younger people reported difficulty finding space to isolate due to shared living arrangements and managing their mental and physical health, middle-aged people reported childcare obligations, and older people perceived themselves as able to take sufficient precautions. Health communication messages attempting to increase compliance with necessary health measures may be strengthened by focusing on and addressing the individual- and institutional-level reasons for noncompliance within particular age groups. The results from our natural language processing analysis of open-ended survey questions demonstrate how researchers and policymakers can rapidly ascertain how their communities are feeling and responding to COVID-19 amid changing conditions.

## Appendix

Multimedia Appendix 1 Supplementary materials.

## Abbreviations

LIWC: Linguistic Inquiry and Word Count
RQ: research question
