# Supplementary material for: Age-Related Differences in Experiences With Social Distancing at the Onset of the COVID-19 Pandemic: A Computational and Content Analytic Investigation of Natural Language From a Social Media Survey
Source: JMIR Hum Factors. 2021 Jun 9;8(2):e26043. doi: 10.2196/26043 (PMC8191726; doi:10.2196/26043)
Supplement: Multimedia Appendix 1 [file humanfactors_v8i2e26043_app1.docx]

**Supplementary Materials**

**Supplement I: LIWC analysis (RQ1)**

For question:

“Tell us how the coronavirus crisis is impacting your life.”

Responses that were analyzed were constructed as a subset of total responses in the following manner:

1. Delete responses for individuals who entered implausible ages of 100 years old (N = 5; these respondents also wrote offensive or irrelevant information in other parts of the survey to suggest that their responses were inappropriate to analyze).
2. Delete the responses for N = 395 participants who did not provide an age
3. Delete responses to this question which were less than 30 words (this is because LIWC works best with responses which are around this size)

The resulting set of responses (N = 6,573) were then analyzed using LIWC, which works by counting the number of words in different language categories in a given text (in this case, each respondent's response to this question) and calculating the percentage of words in each of those categories out of the total number of words in a given text (in this case, in each response). Below are the results of statistical tests (Tukey-Kramer procedure for multiple pairwise comparisons with groups of unequal sample size) of the language categories presented in Figure 1, in which the mean percentage of words in 6 language categories were compared across 4 age groups (1 = 18-31, 2 = 32-44, 3 = 45-64, 4 = 65+).

**Positive emotion terms**

  Tukey multiple comparisons of means

    95% family-wise confidence level

Fit: aov(formula = positive emotion terms ~ agegroup, data = data)

$agegroup

  diff                 lwr                  upr             p adj

2-1 0.21472040  0.03352394 0.3959169 0.0124960

3-1 0.23640067  0.06244982 0.4103515 0.0027147

4-1 0.32243841  0.10912962 0.5357472 0.0005995

3-2 0.02168028 -0.10806658 0.1514271 0.9734489

4-2 0.10771801 -0.07137969 0.2868157 0.4102746

4-3 0.08603773 -0.08572585 0.2578013 0.5711391

**Anxiety-related terms**

  Tukey multiple comparisons of means

    95% family-wise confidence level

Fit: aov(formula = anxiety related terms ~ agegroup, data = data)

$agegroup

 diff               lwr                  upr                   p adj

2-1 -0.1468913 -0.3490226  0.055239954 0.2422628

3-1 -0.2966453 -0.4906939 -0.102596809 0.0005011

4-1 -0.4179998 -0.6559536 -0.180046112 0.0000383

3-2 -0.1497540 -0.2944914 -0.005016679 0.0393034

4-2 -0.2711085 -0.4708986 -0.071318527 0.0027671

4-3 -0.1213545 -0.3129631  0.070254041 0.3631065

**1st person singular pronouns**

  Tukey multiple comparisons of means

    95% family-wise confidence level

Fit: aov(formula = first person singular pronouns ~ agegroup, data = data)

$agegroup

                      diff              lwr                 upr            p adj

2-1 -1.36598339 -1.7914478 -0.94051895 0.0000000

3-1 -1.68293255 -2.0913837 -1.27448137 0.0000000

4-1 -1.44334177 -1.9442087 -0.94247489 0.0000000

3-2 -0.31694916 -0.6216056 -0.01229267 0.0377489

4-2 -0.07735838 -0.4978948  0.34317800 0.9650836

4-3  0.23959078 -0.1637245  0.64290606 0.4215596

**1st person plural pronouns***

  Tukey multiple comparisons of means

    95% family-wise confidence level

Fit: aov(formula = first person plural pronouns ~ agegroup, data = data)

$agegroup

                   diff               lwr               upr              p adj

2-1  1.10893069  0.8212600  1.39660134 0.0000000

3-1  0.84550886  0.5693414  1.12167627 0.0000000

4-1  0.78673958  0.4480868  1.12539232 0.0000000

3-2 -0.26342183 -0.4694102 -0.05743345 0.0056326

4-2 -0.32219111 -0.6065297 -0.03785248 0.0189037

4-3 -0.05876928 -0.3314641  0.21392558 0.9455417

*Our respondents used 1st person plural pronouns (ie, we, us) to refer to a wide variety of different entities (eg, family, friends, the country, the world). Although outside of the scope of our purposes in this paper, further examination of variation in these collective referrents is a fruitful area for future work given the strong connection between individual actions and collective outcomes in the context of the pandemic.

**Family terms**

  Tukey multiple comparisons of means

    95% family-wise confidence level

Fit: aov(formula = family terms ~ agegroup, data = data)

$agegroup

                   diff                 lwr                upr           p adj

2-1  0.44924054  0.25818640  0.64029469 0.0000000

3-1  0.40252720  0.21911284  0.58594157 0.0000001

4-1  0.15000298 -0.07491051  0.37491648 0.3164553

3-2 -0.04671334 -0.18351886  0.09009218 0.8165735

4-2 -0.29923756 -0.48807877 -0.11039635 0.0002754

4-3 -0.25252422 -0.43363232 -0.07141612 0.0019409

**Biological terms**

  Tukey multiple comparisons of means

    95% family-wise confidence level

Fit: aov(formula = biological terms ~ agegroup, data = data)

$agegroup

                      diff                 lwr            upr              p adj

2-1 0.003917856 -0.29099793 0.2988336 0.9999857

3-1 0.139968536 -0.14315429 0.4230914 0.5818920

4-1 0.632867041  0.28568515 0.9800489 0.0000170

3-2 0.136050680 -0.07512562 0.3472270 0.3475989

4-2 0.628949184  0.33744935 0.9204490 0.0000002

4-3 0.492898505  0.21333568 0.7724613 0.0000354

Below is the paragraph from the Language and the Impact of the COVID-19 Pandemic section of the main text modified to include the *P*-values from the relevant age group comparisons:

However, these overall patterns hide significant differences between age groups. As Figure 1 shows, younger people (18-31) were more anxious (greater usage of anxiety-related terms: *P_18-31 vs. 32-44_* = .24, *P_18-31 vs. 45-64_* < .001, *P_18-31 vs. 65+_* < .001), less emotionally positive (lesser usage of positive emotion terms: *P_18-31 vs. 32-44_* = .01, *P_18-31 vs. 45-64_* = .002, *P_18-31 vs. 65+_* < .001), self-focused (greater use of 1st person singular pronouns: *P_18-31 vs. 32-44_* < .001, *P_18-31 vs. 45-64_* < .001, *P_18-31 vs. 65+_* < .001), and less concerned with family (lesser use of family-related terms: *P_18-31 vs. 32-44_* < .001, *P_18-31 vs. 45-64_* < .001, *P_18-31 vs. 65+_* = .32), while middle-aged people were group oriented (32-44; greater use of 1st person plural pronouns: *P_32-44 vs. 18-31_* < .001, *P_32-44 vs. 45-64_* = .006, *P_32-44 vs. 65+_* = .02) and focused on family (32-64; greater use of family-related terms: *P_32-44 vs. 18-31_* < .001, *P_32-44 vs. 65+_* < .001, *P_45-64 vs. 18-31_* < .001, *P_45-64 vs. 65+_* = .001). Unsurprisingly, the oldest and most at-risk group (65+) wrote frequently about biological terms (e.g., health-related topics) (greater use of biological terms: *P_65+ vs. 18-31_* < .001, *P_65+ vs. 32-44_* < .001, *P_65+ vs. 45-64_* < .001) but were surprisingly low in anxiety (lesser use of anxiety-related terms: *P_65+ vs. 18-31_* < .001, *P_65+ vs. 32-44_* = .002, *P_65+ vs. 45-64_* = .36) and emotionally positive (greater use of positive emotion terms: *P_65+ vs. 18-31_* < .001, *P_65+ vs. 32-44_* = .41, *P_65+ vs. 45-64_* = .57) relative to those at lower risk (all *P* values corrected for multiple comparisons).

Below is a detailed description of the analyses reported in the Discussion section re: the size of the positivity bias in older adults’ emotions (page 21):

Pennebaker and Stone (2003) analyzed text samples from disclosure studies from over 3,000 research participants from 45 different studies representing 21 laboratories in 3 countries and investigated the association between language use and age. They also used LIWC, the same methodological tool we used to analyze participants’ language. In their Figure 1, they provide estimates of the proportion of total words that are positive emotion words in their dataset. While they do not use the same age groupings as we do in our analysis, we can transform their age categories into ones closer to ours in order to make comparisons between our findings regarding older adults’ greater usage of positive emotion terms. Specifically, we transformed Pennebaker and Stone (2003)’s six age groups of 8-14, 15-24, 25-39, 40-54, 55-69, 70+ to four groups: 8-24, 25-39, 40-54, 55+, which we can then compare to our four age groups of 18-31, 32-44, 45-64, 65+. From there, we can compare the relative magnitudes of the positivity biases between our results and Penenbaker and Stone’s data by subtracting the % of total words that are positive emotion words in our respective oldest age groups from our youngest age groups (in the case where Pennebaker and Stone’s original age groups were combined, we averaged the positive emotion words score in order to arrive at the combined group’s positive emotion words score). In our data, that difference is equal to 0.32 and in Pennebaker and Stone’s data that difference is equal to approximately 0.975 (this is an approximation as these values are only reported visually in Figure 1). Therefore, it appears that the magnitude of the positivity bias between older and younger adults is about 3 times larger in magnitude in Pennebaker and Stone’s large corpus of “non-COVID” language than in our COVID-related language.

(Schwartz et al., 2013) analyzed Facebook messages from tens of thousands of volunteers and investigated variations in language across personality, gender, and age. We worked with one of the paper’s authors to perform an analysis of their messages with LIWC that would allow us to make comparisons between the age-related sentiment patterns in our COVID-related language and age-related sentiment patterns in their “non-COVID” language. Given the younger skew of their Facebook messages sample (*M_age_* = 23.43, *SD_age_* = 8.96), we were only able to examine sentiment in individuals up to 60 years of age in the Facebook language. Therefore, the age buckets for the Facebook messages data are 18-31, 32-44, and 45-60. We re-analyzed our survey responses using those same 3 age groups in order to compare sentiment between older and younger individuals. In Schwartz et al.’s data, the mean difference in percentage of total words that are positive emotion words between the oldest (45-60) and youngest (18-31) groups is 0.28%. In our data (comparing between these two age groups), the difference is 0.23%. The difference in our data is significantly smaller than the difference reported in Schwartz et al.’s data, *t*(50,723) = -9.49, *P* < .001.

In addition to positive emotion words, we also examined anxiety-related words, which in our data appear to be used significantly more by younger than older individuals (see Figure 1). Performing the same analysis as was just described, except this time on anxiety-related words rather than positive emotion words, reveals a difference in usage of anxiety words between younger and older individuals in Schwartz et al.’s data of 0.04 while that same difference in our data is 0.33. This suggests that the gap in anxiety-related word usage between younger and older individuals in our COVID-related language data was significantly larger (about 7.9 times) than in the non-COVID Facebook messages, *t*(47,676) = 200.96, *P* < .001.

**Supplement II: Topical Analysis of Pandemic Impact by Age Group with Meaning Extraction Method (RQ1)**

To further explore the impact of the pandemic on people’s lives, we identified themes in language use for each age group by using the meaning extraction method, which relies on principal-components analysis of content words in language corpuses (Markowitz, 2021). Examination of KMO measures of sampling adequacy (KMO = .59 - .68) and Bartlett’s tests of sphericity (X2 = 10,210 – 23251, p < .001) suggested the language data by age group were suitable for PCA. Data were pre-processed using the Meaning Extraction Helper software, to remove function words (i.e., prepositions) and words with low base rates (present in < 5% of responses), and calculate whether content words (i.e., nouns, verbs) were present (coded as “1”) or absent (coded as “0”) within a response. This process yielded a series of 93 unique unigrams of content words.

According to processes discussed in Markowitz (2021), we treated the four sets of open-ended responses to the question “Tell us how the coronavirus crisis is impacting your life,” by age group as four corpuses of language to be analyzed. Each corpus was subjected to a separate principal-component analyses (PCAs). Unspecified extraction of components yielded a large *n* of components (> 20) for each age group, which was difficult to parse. As a result, we specified the PCA to extract five factors.

Each age group yielded five components. Content words (i.e., grocery, distance) were retained for each factor within the age group if the factor loadings were greater than or equal to the absolute value of .30. The research team discussed the interpretation of components to create labels for each factor by age group. The results of the PCA analyses can be found in Table 1.

**Supplement III: Thematic Content Analysis (RQ2)**

We were also interested in understanding the reasons why people were not complying with social distancing recommendations. Participants were asked to respond, “yes” or “no” to whether they were social distancing and self-isolating as much as possible. If they selected “yes,” they were asked to select from a set of options (“I cannot afford to miss work”, “I do not have sufficient space to self-isolate”, “I do not believe social isolation is effective at preventing the spread of COVID-19”, “I have to attend class in-person”) with the option to also write-in their own response.

To analyze these open-ended responses, we used qualitative methods of thematic content analysis to identify, analyze, and report the key themes guiding individuals’ responses. This process was conducted by two independent raters. First, the first coder reviewed the entire dataset and generated a list of six preliminary themes regarding participants’ rationale for non-compliance. Upon further review and analysis with the research team, these codes were updated and finalized in a codebook.

Noting that many participants’ responses described behaviors that were compliant with social distancing and social isolation recommendations, we created a code to capture compliance (coded as “1”) and non-compliance (coded as “2”). Analysis of written responses revealed that approximately a third (*N* = 3,652) were in fact in compliance with recommended health guidances (i.e., only leaving their house to buy groceries, find essential supplies, or attend necessary medical appointments), and these participants were coded as compliant.

The second category of non-compliance was then explored further in a secondary code labeled “reasons for non-compliance.” Iterative review through the content analytic process yielded six key themes of non-compliance [See Table 2 below for descriptions and examples]. The first coder reviewed the entire dataset and the second coder reviewed 25% of the codes, with good inter-rater reliability (*Cohen’s kappa, compliance* = .81; *Cohen’s kappa, non-compliance reasons* = .76). The codebook below illustrates the criteria used by both coders.

| **Theme** | **Description** | **Example** |
| --- | --- | --- |
| Mental and Physical Health | Participants are not fully compliant because of concerns related to their mental and physical health.    This may mean:    (1) Engaging in physical, social, or routine activities to take care of mental health issues exacerbated or caused by following social isolation policies (i.e., developing “cabin fever,” depression or boredom from staying indoors)   (2) Engaging in indoors or outdoors exercise for their physical health in a manner that *does not comply* with social isolation policies (i.e., attending a dance class, going to the gym, playing sports with others). Exercise that follows guidelines is coded as “Compliant” (i.e., walking dogs, going on solo hikes) because they are not violating any policies. | “Staying in my home 24 hours of every day is depressing”    “I have to get outside now and then for my own sanity”    “Exercise is vital to mental/physical health”    “Total self isolation would probably drive me to suicide” |
| Children/childcare | Participants are not fully compliant because of concerns relating to childcare, including  (1) Taking their children outdoors or to social events for both children’s and parents’ well-being  (2) Continuing interactions with others who provide or need childcare (i.e., parents working-from-home)  (3) Transporting children to and from school (if they are still open) | “I have kids and it’s impossible to keep them grounded all the time”  “Custody rules - have to physically meet at a public space to exchange the child.” |
| I cannot afford to miss work | Participants are not fully compliant because of work commitments:    (1) Serving as an essential worker.  (2) Employers do not have full work-from-home policies or are requiring employees to continue coming to work  (3) They risk losing their job, insurance, benefits, or PTO if they do not go to work in line with social isolation policies  (4) They are managing a business in a non-essential industry that they need to continue for income (i.e., food and restaurants, tourism)  (5) They must continue to work to secure a source of income for their families  (6) Other members of their household are continuing to work, thereby increasing the risk of exposure to the family | “Work is not canceled, if I don’t go I’ll lose my job”    “My employer refuses to close down.”  “Work is not allowing WFH unless we are sick.”  “I go to work 2 days a week and work remotely 3 days a week”    “I have a business to run and clients need me”    “Other household members have work outside the house” |
| Society is over-reacting | Participants are not fully compliant because they believe that the recommended social isolation policies are an over-reaction to the COVID19 outbreak, including:    (1) They believe there is no threat of COVID19 exposure in their *local community*  (2) The *normative beliefs* of others in their life (i.e. household members, friends) view compliance as an over-reaction  (3) They are not concerned about COVID19 until it affects them personally, or do not believe it will affect them personally  (4) They believe that the recommended policies to contain COVID19 are disproportionate to its risk | “I haven’t yet bought into the paranoia”  “I don’t believe it’s a realistic way of life unless you are diagnosed with the virus”  “Cavalier attitude about my body’s response to virus”  “I’m working and I have a great immune system” |
| Non-essential activities | Participants are not fully compliant because they want to engage in non-essential activities such as:    (1) Having social gatherings or interactions with friends, family, or community members  (2) Completing non-essential errands and activities (i.e. going shopping for clothes, playing sports)  (3) Continuing to attend that have made online versions available (i.e., religious services)  (4) Wanting to avoid significant disruption to personal routines and everyday life  (5) Supporting the economy, such as eating at small businesses | “We still see friends and family in small groups at our home or theirs”  “This is not serious enough worth changing my lifestyle over.”  “Some appointments are in-person. Need to see friends sometimes.”    “Social needs, shopping needs”    “We have to keep living. Life has to go on”    “Still going to restaurants, want to support businesses” |
| Taking Sufficient Precautions | Participants are not fully compliant because they believe the actions they are already taking to protect themselves from COVID19 are sufficient, and thus they do not need to follow social isolation policies, such as:    (1) Following some, but not all of the recommended policies (i.e, keeping 6 feet away from others but still going out to eat)  (2) Utilizing personal “common sense” and caution instead of following social isolation policies  (3) Making no changes to their lifestyle and habits because they perceive themselves as already clean, cautious, or socially isolated | “Use normal hand washing, and practice good hygiene. Then there is nothing to worry about!”  “Protecting myself by using hand sanitizer, personal hygiene, and common sense.”    “I want to maintain some normalcy while using precautions”    “I’m generally cautious”    “I’m still going to go to the gym. I’m going to wash my hands more and the equipment, but I don’t plan to miss, I have a competition.” |

**Supplement IV: Additional Information about Participant Recruitment**

This study was approved by the authors’ university’s IRB. All participants were informed of the study goals and provided informed consent prior to participating in the survey. The consent form was integrated into the online survey instrument. No personally identifying information was collected from participants. The social media posts recruiting individuals to participate in the survey provided a brief description of the purposes of the study and described eligibility criteria (i.e., must be at least 18 years of age to participate). Example text from one recruitment message was: “(University name removed) study to evaluate Coronavirus epidemic. Please spend 5mins to help us track the impact of the virus on the community and understand the actions individuals and households are taking in response to the epidemic. You must be 18+yr to participate. (Link to survey)”.

Our final survey instrument, which contained 21 questions, was based upon the results of pilot testing larger surveys with paper questionnaires. To construct the survey, we came together as a team and decided on the topics that were most urgent/interesting/useful to the public. As this survey was created and distributed in the early days of the pandemic when community spread had just begun to grow in the United States, there were not many resources available regarding COVID-specific questions. Additionally, given that our survey would be distributed widely on social media, we wanted it to be less than 5 minutes in length so that a large number of individuals would take it. After timing initial versions of the paper questionnaires, we cut the larger body of questions into our final set of 21 based on optimizing for 1) what was important and urgent to know and 2) length. We also wanted a mixture of closed- and open-ended questions. We collectively decided on phrasing of specific questions and wherever possible used validated instruments (e.g., self-assessed health). Nelson et al. (2020) includes further details about survey design and distribution.

**References**

Markowitz, D. M. (2021). The Meaning Extraction Method: An Approach to Evaluate Content

Patterns from Large-Scale Language Data. *Frontiers in Communication*, *6*. https://doi.org/10.3389/fcomm.2021.588823

Nelson, L. M., Simard, J. F., Oluyomi, A., Nava, V., Rosas, L. G., Bondy, M., & Linos, E.

(2020). US Public Concerns About the COVID-19 Pandemic From Results of a Survey Given via Social Media. *JAMA Internal Medicine*, *180*(7), 1020–1022. https://doi.org/10.1001/jamainternmed.2020.1369

Pennebaker, J. W., & Stone, L. D. (2003). Words of wisdom: Language use over the life span. *Journal of Personality and Social Psychology, 85(2), 291–301*. https://doi.org/10.1037/0022-3514.85.2.291

Schwartz, H. A., Eichstaedt, J. C., Kern, M. L., Dziurzynski, L., Ramones, S. M., Agrawal, M.,

Shah, A., Kosinski, M., Stillwell, D., Seligman, M. E. P., & Ungar, L. H. (2013). Personality, Gender, and Age in the Language of Social Media: The Open-Vocabulary Approach. *PLOS ONE*, *8*(9), e73791. https://doi.org/10.1371/journal.pone.0073791
